# Association between aromatase in human brains and personality traits

**DOI:** 10.1038/s41598-018-35065-4

**Published:** 2018-11-15

**Authors:** Kayo Takahashi, Takamitsu Hosoya, Kayo Onoe, Tadayuki Takashima, Masaaki Tanaka, Akira Ishii, Yasuhito Nakatomi, Shusaku Tazawa, Kazuhiro Takahashi, Hisashi Doi, Yasuhiro Wada, Yasuyoshi Watanabe

**Affiliations:** 1 RIKEN Center for Life Science Technologies, 6-7-3 Minatojima-minamimachi, Chuo-ku, Kobe, Hyogo 650-0047 Japan; 2 RIKEN Center for Biosystems Dynamics Research, 6-7-3 Minatojima-minamimachi, Chuo-ku, Kobe, Hyogo 650-0047 Japan; 3 Department of Physiology, Osaka City University Graduate School of Medicine, 1-4-3 Asahi-cho, Abeno-ku, Osaka 545-8585 Japan; 4 Institute of Biomaterials and Bioengineering, Tokyo Medical and Dental University, (TMDU), 2-3-10 Kanda-Surugadai, Chiyoda-ku, Tokyo 101-0062 Japan; 5 Department of Metabolism, Endocrinology and Molecular Medicine, Osaka City University Graduate School of Medicine, 1-4-3 Asahi-cho, Abeno-ku, Osaka, 545-8585 Japan

## Abstract

Aromatase, an enzyme that converts androgens to estrogens, has been reported to be involved in several brain functions, including synaptic plasticity, neurogenesis, neuroprotection, and regulation of sexual and emotional behaviours in rodents, pathophysiology of Alzheimer’s disease and autism spectrum disorders in humans. Aromatase has been reported to be involved in aggressive behaviours in genetically modified mice and in personality traits by genotyping studies on humans. However, no study has investigated the relationship between aromatase in living brains and personality traits including aggression. We performed a positron emission tomography (PET) study in 21 healthy subjects using ^11^C-cetrozole, which has high selectivity and affinity for aromatase. Before performing PET scans, subjects answered the Buss-Perry Aggression Questionnaire and Temperament and Character Inventory to measure their aggression and personality traits, respectively. A strong accumulation of ^11^C-cetrozole was detected in the thalamus, hypothalamus, amygdala, and medulla. Females showed associations between aromatase levels in subcortical regions, such as the amygdala and supraoptic nucleus of the hypothalamus, and personality traits such as aggression, novelty seeking, and self-transcendence. In contrast, males exhibited associations between aromatase levels in the cortices and harm avoidance, persistence, and self-transcendence. The association of aromatase levels in the thalamus with cooperativeness was common to both sexes. The present study suggests that there might exist associations between aromatase in the brain and personality traits. Some of these associations may differ between sexes, while others are likely common to both.

## Introduction

Aromatase is an enzyme that converts androgens to estrogens and is localized not only in the gonads but also in the brain^1^. In mammals such as rodents and non-human primates, the regions rich in aromatase are the hypothalamus and amygdala^2–4^. In addition, the thalamus also contains high concentrations of aromatase in humans^5–7^. Aromatase in the brain has been suggested to be related to several brain functions. Aromatase knockout mice display altered aggressive behaviours^8–11^, disrupted sexual behaviour^9,11^, and displayed depressive-like behaviour^12^. Human postmortem brain studies demonstrated lower aromatase expression in the hypothalamus of Alzheimer’s disease^13^ and depression patients^14^, and in the frontal cortex of autism spectrum disorder (ASD) patients^15,16^, as compared with the same brain regions of control subjects.

The association between aromatase and aggression in animals has been reviewed by Trainor *et al*.^17^. Rodent studies suggested that the medial amygdala has been related to aggression^18–20^. In human studies, fMRI studies showed increased amygdala activation related to aggression^21,22^ and MRI morphometric study demonstrated reduced amygdala volume in healthy volunteers with higher aggression trait^23^. The lines of evidence indicate that the amygdala may be a key region in aggression. Besides aggression, there is a study indicating a relation between aromatase gene polymorphism and personality trait, harm avoidance^24^. So far, however, there was no study on the association between aromatase in living brain and personality traits including aggression. For assessment of personality traits, a questionnaire method is often used. In this study, we employed the Buss-Perry Aggression Questionnaire (BAQ)^25,26^ for aggression assessment and the Temperament and Character Inventory (TCI)^27,28^, which can evaluate personality traits, namely, novelty seeking, harm avoidance, reward dependence, persistence, self-directedness, cooperativeness, and self-transcendence.

To investigate molecular dynamics in the living body, positron emission tomography (PET) is a suitable technique, which allows quantitative analysis of compound accumulation in tissues. Previously, a PET study using ^11^C-vorozole, which was developed as the first PET probe for aromatase imaging, was performed to image aromatase in the brain of healthy men and women^6,7^. In that study, the authors demonstrated a unique distribution of aromatase in living human brains for the first time. However, a certain amount of nonspecific signals was observed in ^11^C-vorozole PET images probably caused by unintended reuptake of the radioactive metabolite into the brain.

We have developed a novel PET probe for aromatase imaging to overcome the disadvantages of ^11^C-vorozole, ^11^C-cetrozole^29^. Our previous study showed that ^11^C-cetrozole had a higher signal-to-noise ratio than ^11^C-vorozole since almost no radioactive metabolite of ^11^C-cetrozole was not taken up into the brain, that is, ^11^C-cetrozole is superior to ^11^C-vorozole in terms of specificity and metabolic stability^29^. In the present study, we performed a ^11^C-cetrozole PET study in healthy subjects to examine the association between aromatase in the brain and personality traits.

## Results

### Distribution of aromatase in human brain

**Figure 1:**
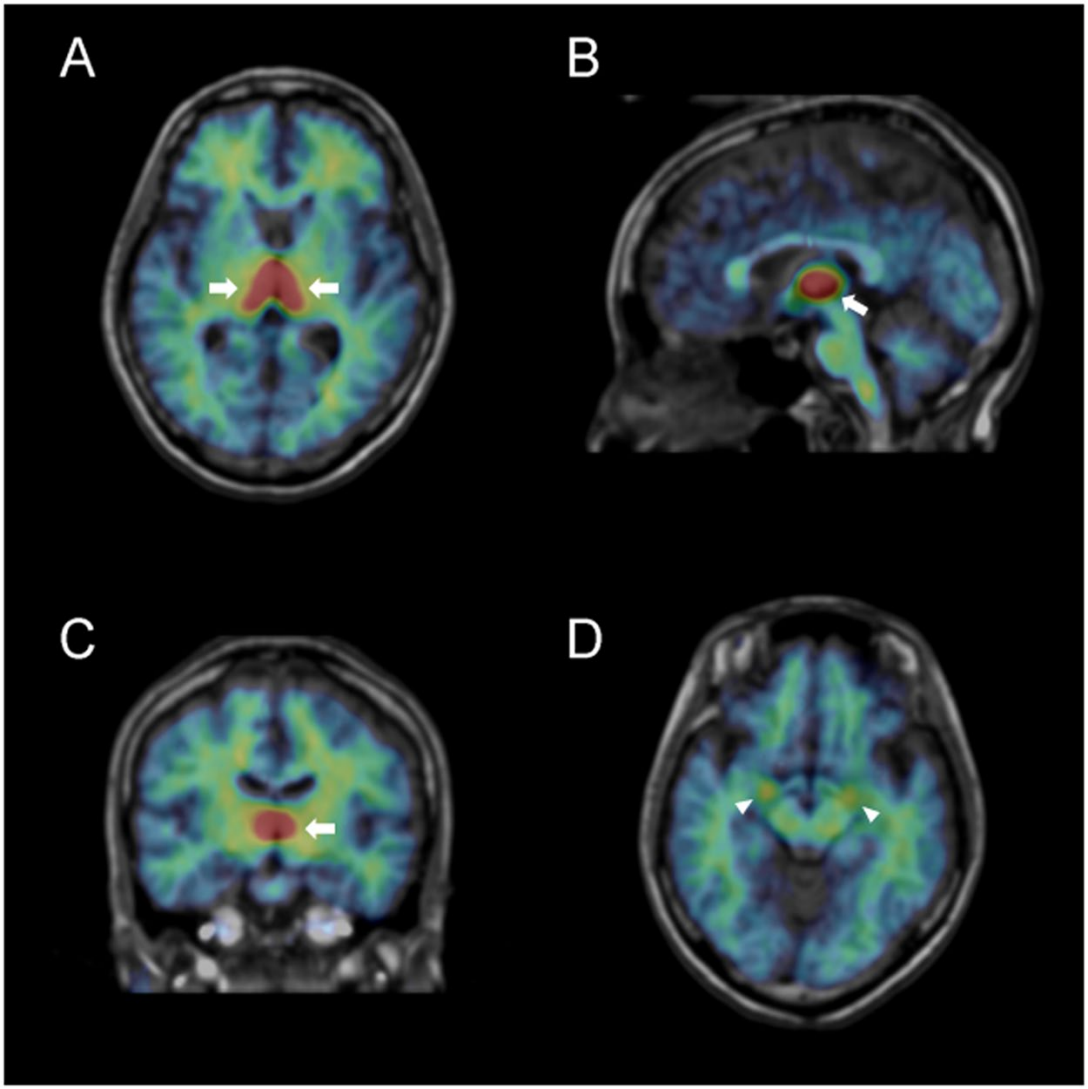
Representative distribution volume (BP_ND_ + 1) images of ^11^C-cetrozole in a female human brain (rainbow colour scale) superimposed on the structural MR image (gray scale) of the same subject. (**A**) transaxial slice at level of the thalamus; (**B**) sagittal slice at the midline; (**C**) coronal slice at the level of the thalamus; (**D**) transaxial slice at the level of the amygdala. Arrows and arrow heads indicate the thalamus and amygdala, respectively.

**Figure 2:**
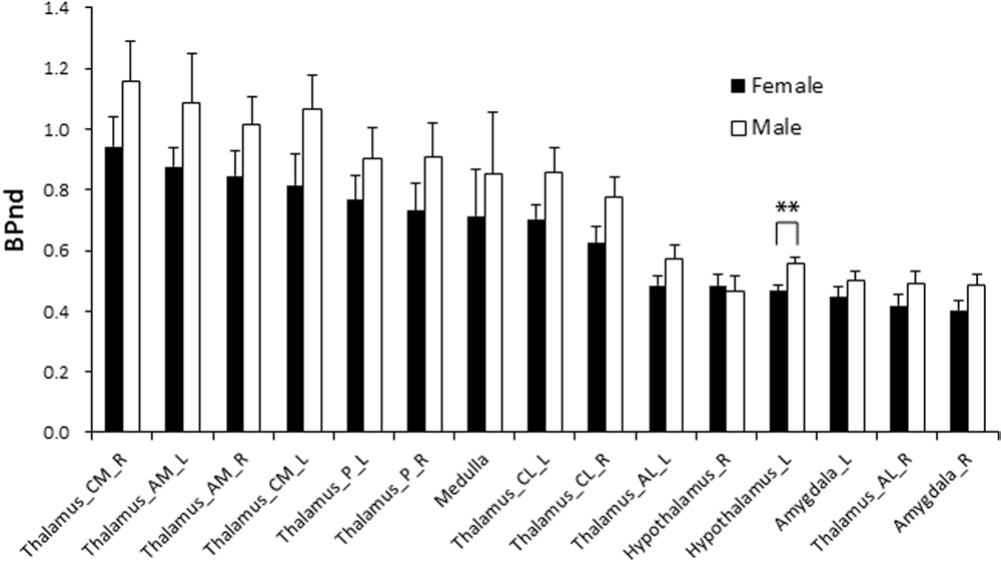
BP_ND_ values for the subregions of thalamus, amygdala, hypothalamus, and medulla. In all regions except for the right hypothalamus, males had higher BP_ND_ values than females. A significant sex difference was observed only in the left hypothalamus (**P = 0.005). AM, anterior medial; AL, anterior lateral; CM, central medial; CL, central lateral; P, posterior; L, left; R, right.

The binding potential (BP_ND_) values of ^11^C-cetrozole, which are an index of aromatase concentration, were calculated in 21 healthy individuals (10 females and 11 males). High BP_ND_s were found in the thalamus with heterogeneous distribution among the medulla, hypothalamus, and amygdala in both sexes (Figs 1 and 2). Males had higher BP_ND_ values in most of these regions than females, except for in the right hypothalamus; however, a significant sex difference was found only in the left hypothalamus (P = 0.005; Fig. 2). There were no regions which BP_ND_s depended on sex hormone levels in plasma (estradiol for females, and free testosterone for males).

### Association between aromatase and personality traits

**Figure 3:**
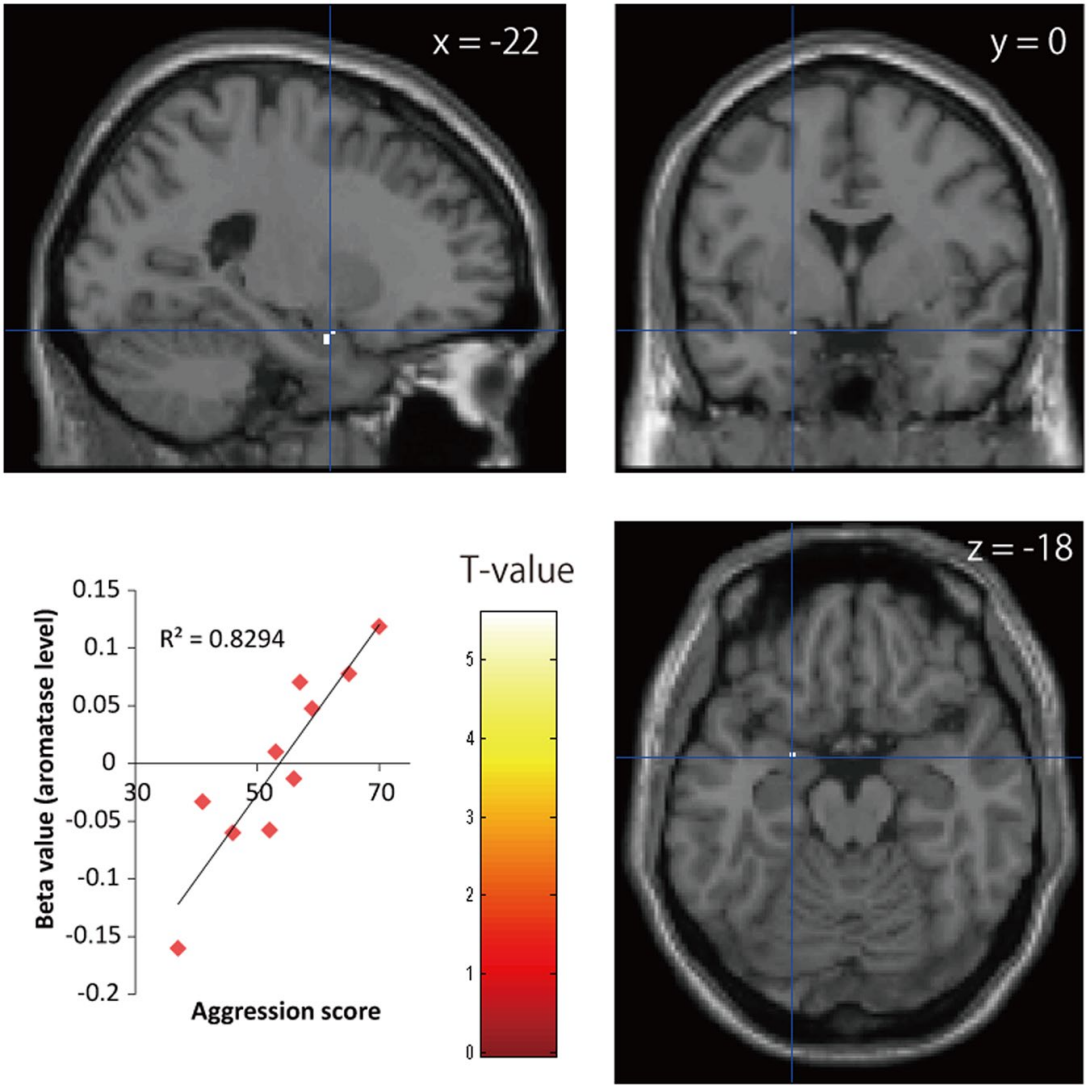
Statistical parametric maps of associations between ^11^C-cetrozole BP_ND_ values in the amygdala of females and aggression scores (P_FWE-corr_ < 0.05, 40 mm^3^). Peak coordinates (x = −22, y = 0, z = −18) are mapped on the template brain.

**Figure 4:**
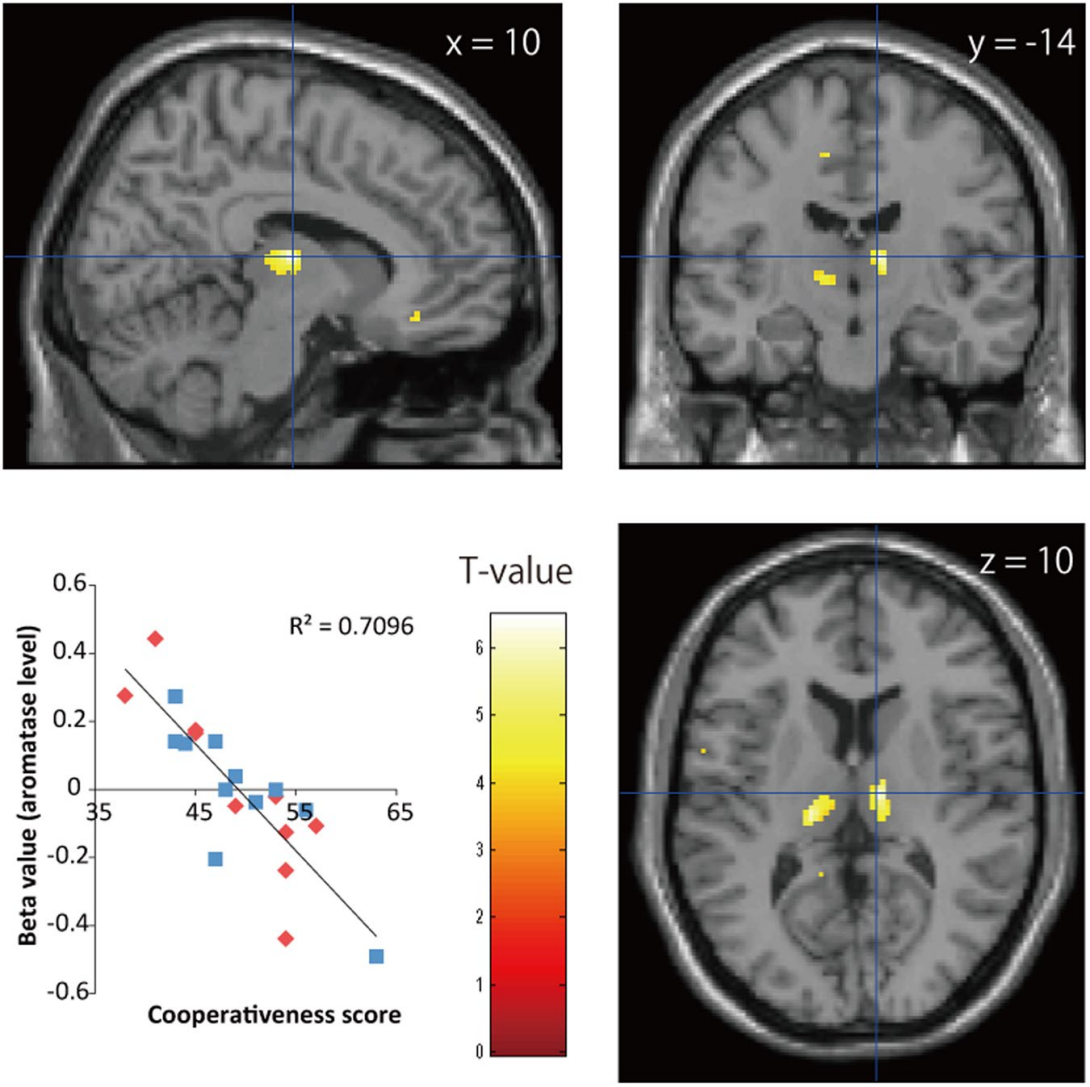
Statistical parametric maps of associations between ^11^C-cetrozole BP_ND_ values in the thalamus and cooperativeness scores in females and males (P < 0.001, uncorrected, 640 mm^3^). Peak coordinates (x = 10, y = −14, z = 10) are mapped on the template brain. Red diamonds and blue squares represent the data from females and males, respectively.

We assessed associations between BP_ND_ images of ^11^C-cetrozole and scores of BAQ. Given that earlier studies showed that the amygdala is implicated in aggression^18–23^, we focused on the amygdala as a volume of interest (VOI). Using Statistical Parametric Mapping 8 software (SPM8, Wellcome Department of Imaging Neuroscience), a voxel-wise analysis corrected by family-wise error rate for aggression scores on the VOI was performed. Apparently, region-specific differences among individuals are evident and the associations between personality traits and region-specific level of aromatase were also observed (Figs 3 and 4). In females, aggression scores were positively associated with BP_ND_ in the left amygdala (P_FWE-corr_ < 0.05, R^2^ = 0.83, Fig. 3). In contrast, data from male and combined data from male and female did not exhibit a significant association between aggression scores and BP_ND_ in the amygdala. For an evaluation of individuals’ personality traits, subjects answered TCI. Associations between traits and brain regions analysed in each sex group are listed in Table 1. Concerning other traits, sex-specific associations were observed in the amygdala and supraoptic nucleus of hypothalamus and in the inferior parietal gyrus in females. On the other hand, males-specific associations were observed in the anterior cingulate gyrus, supramarginal gyrus, caudate nucleus, pons, and midbrain. When the data of female and male were combined, almost no significant associations were found, except for cooperativeness. Cooperativeness scores were negatively associated with BP_ND_ in the bilateral thalamus of females and males (R^2^ = 0.71, Fig. 4). The subregions that associated with cooperativeness were localized to the ventral lateral and ventral posterior parts of the thalamus.

**Table 1:** The association between ^11^C-cetrozole BP_ND_ and traits (scores on TCI; P < 0.001, uncorrected, cluster size ≥ 80 mm^3^).

Traits	Association	Sex	Region	Side	MNI Coordinates			P value	Z score	Cluster size (mm^3^)
					x	y	z			
Novelty Seeking	Negative Negative Positive	F F M	Hypothalamus(SON) Amygdala Caudate nucleus	R R R	8 24 8	2 −6 18	−14 −20 −4	<0.001 <0.001 <0.001	4.04 3.71 3.96	136 80 104
Harm Avoidance	Negative Negative	M M	Pons Supramarginal gyrus	R R	10 60	−22 −44	−30 34	<0.001 <0.001	3.55 3.38	88 88
Reward Dependence	Positive	M	Thalamus	L	−18	−26	6	<0.001	3.79	152
Persistence	Negative Negative Negative	M M F & M	Anterior cingulate gyrus Supramarginal gyrus Lingual gyrus	L R L	−12 58 −16	42 −46 −76	14 38 −12	<0.001 <0.001 <0.001	3.76 3.43 3.54	96 96 144
Self-Directedness	Positive	F	Inferior parietal gyrus	L	−36	−56	46	<0.001	3.58	96
Cooperativeness	Positive Negative Negative Negative Negative Negative	M F & M F & M F & M F & M F & M	Anterior cingulate gyrus Thalamus Thalamus Superior frontal gyrus Inferior frontal gyrus, triangular part Inferior frontal gyrus, orbital part	L R L L L L	−12 10 −16 −22 −48 −44	40 −14 −24 34 28 24	20 10 8 38 24 −6	<0.001 <0.001 <0.001 <0.001 <0.001 <0.001	3.94 4.17 3.99 3.87 3.76 3.76	104 640 1296 96 168 160
Self-Transcendence	Positive Negative Negative	F M M	Hypothalamus (SON) Anterior cingulate gyrus Midbrain	R L R	8 −12 8	2 40 −26	−14 20 −18	<0.001 <0.001 <0.001	4.01 4.02 3.70	120 152 80

**Figure 5:**
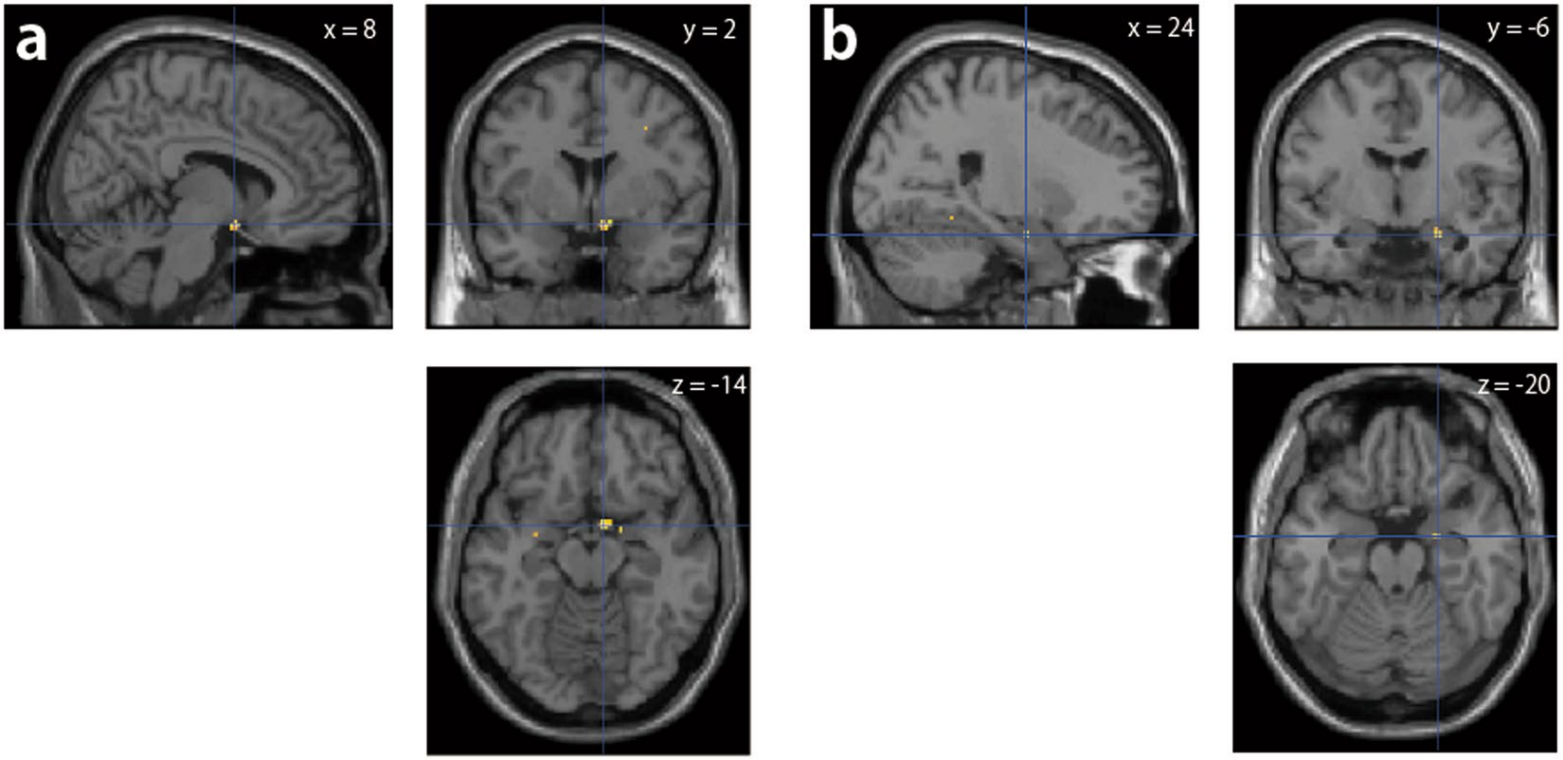
Two regions that exhibited associations between BP_ND_ of ^11^C-cetrozole and female traits are the right supraoptic nucleus of the hypothalamus ((**a**) SON; MNI: x = 8, y = 2, z = −14) and the right amygdala ((**b**) x = 24, y = −6, z = −20).

There were 2 regions that exhibited associations between BP_ND_ of ^11^C-cetrozole and female traits, namely, the right supraoptic nucleus of the hypothalamus (SON; MNI: x = 8, y = 2, z = −14) and the right amygdala (x = 24, y = −6, z = −20), although several associations were present in small clusters (Tables 2 and 3, Fig. 5). The traits that associated with BP_ND_ in the SON were novelty seeking (negative, R^2^ = 0.998), harm avoidance (positive, R^2^ = 0.97), reward dependence (negative, R^2^ = 0.997), persistence (negative, R^2^ = 0.98), cooperativeness (positive, R^2^ = 0.995), and self-transcendence (positive, R^2^ = 0.997; Table 2). The traits associated with BP_ND_ in the right amygdala were novelty seeking (negative, R^2^ = 0.999), persistence (negative, R^2^ = 0.994), cooperativeness (positive, R^2^ = 0.999), and self-transcendence (positive, R^2^ = 0.999; Table 3).

**Table 2:** Traits in females that associated with BP_ND_ of ^11^C-cetrozole in the SON (x = 8, y = 2, z = −14). No traits in males were associated with BP_ND_ in this region.

Traits	Association	P value	Z score	Cluster size (mm^3^)
Novelty Seeking	Negative	<0.001	4.04	136
Harm Avoidance	Positive	<0.001	3.43	16
Reward Dependence	Negative	<0.001	3.06	56
Persistence	Negative	<0.001	3.58	24
Cooperativeness	Positive	<0.001	3.62	32
Self-Transcendence	Positive	<0.001	4.01	120

**Table 3:** Traits in females that associated with the BP_ND_ of ^11^C-cetrozole in the right amygdala (x = 24, y = −6, z = −20). No traits in males were associated with this region.

Traits	Association	P value	Z score	Cluster size (mm^3^)
Novelty Seeking	Negative	<0.001	3.71	80
Persistence	Negative	<0.001	3.40	24
Cooperativeness	Positive	<0.001	3.44	32
Self-Transcendence	Positive	<0.001	3.50	40

## Discussion

In this study, we demonstrated the distribution of aromatase in living human brains using our originally developed PET probe, ^11^C-cetrozole, and suggested that aromatase levels in the brain may relate to personality traits. The first PET scan of brain aromatase using ^11^C-vorozole was performed by Biegon *et al*.^6,7^. They demonstrated high levels of aromatase in the thalamus, amygdala, preoptic area, medulla, etc. ^11^C-Vorozole had high specificity and affinity for aromatase; however, the metabolites of ^11^C-vorozole were taken up into the brain with radiolabelling^29^. Thus, the measurements were less quantitative. Aiming for a more quantitative measurement of aromatase, we developed ^11^C-cetrozole as a novel PET probe for aromatase imaging^29^.

In the present study, we performed PET scans with ^11^C-cetrozole in 21 healthy human subjects. Approximately 50% of parental compound was intact 60 min after injection, indicating that this PET probe was suitable to measure aromatase in a living body (Supplemental Fig. S1). High BP_ND_s of ^11^C-cetrozole were found in the thalamus with a heterogeneous distribution among the amygdala, hypothalamus, and medulla in both sexes (Figs 1 and 2). The distribution pattern of ^11^C-cetrozole binding was consistent with that of ^11^C-vorozole^7^ and with immunohistochemistry in the postmortem human brain^5^, suggesting that considerable aromatase enzyme is expressed in the thalamus, hypothalamus, amygdala, and medulla in the human brain. Unlike other mammals that have more aromatase enzyme in male brains, such as rats and monkeys^2–4^, there was no distinct sex difference in aromatase levels in our human cohort, except for in the left hypothalamus (P = 0.005). However, males showed a tendency towards relatively higher aromatase expression in all brain regions than females, except for the right hypothalamus. The reason why males have more aromatase in the brain than females do is considered to compensate lower circulating estrogens since estrogen is an important hormone related to regulation of sexual behaviour and emotions, neural plasticity, neuroprotection, etc. in the brain^30–32^. In the present study we showed that BP_ND_ of ^11^C-cetrozole varied between individuals, especially in the thalamic subregions. This variability was associated with personality trait variability.

Both females and males showed a negative association between BP_ND_ of ^11^C-cetrozole and cooperative scores in the thalamus region in this study. The subregions that associated with cooperativeness were localized to the ventral lateral and ventral posterior parts of the thalamus. The ventral lateral nucleus of the thalamus contains estrogen receptor β and is known to project to the primary motor cortex^33^. Diffusion-weighted imaging studies have segmented the thalamus on a connectivity basis and reported individual variations in segmentation^34^. Johansen-Berg *et al*.^34^ reported that the ventral lateral nucleus primarily connected to the prefrontal cortex in some subjects and to the primary motor cortex in others. This individual variation may provide cause of different personality. High aromatase in the thalamus is unique to humans, while monkeys, baboons, and rats have high amount of aromatase in the amygdala and hypothalamus^3,4,7,29,35,36^. The characteristically high social abilities of humans such as cooperativeness may be processed in the thalamus through the regulation of estrogens.

In addition to cooperativeness and the thalamus, the traits and associated regions were different between sexes (Table 1). In females, sex-specific associations were observed in the amygdala and supraoptic nucleus of hypothalamus and in the inferior parietal gyrus. On the other hand, males-specific associations were observed in the anterior cingulate gyrus, supramarginal gyrus, caudate nucleus, pons, and midbrain.

The right SON and the right amygdala showed associations between BP_ND_s of ^11^C-cetrozole and female traits (Fig. 5). The localization of aromatase in the SON was demonstrated previously via immunohistochemistry^13^. Further, the SON is known to be a region in which oxytocin is synthesized^37^. Oxytocin production is regulated by estradiol^38^. Regulation of oxytocin by estradiol may affect personality traits given that oxytocin is implicated in stress susceptibility, emotion, memory, and social interaction^39^. The traits that associated with BP_ND_ in the SON were novelty seeking (negative), harm avoidance (positive), reward dependence (negative), persistence (negative), cooperativeness (positive), and self-transcendence (positive; Table 2). The traits associated with BP_ND_ in the right amygdala were novelty seeking (negative), persistence (negative), cooperativeness (positive), and self-transcendence (positive; Table 3). These 4 traits are consistent with the traits that associated with BP_ND_ in the SON, which suggests functional connectivity between the right amygdala and SON in females.

Since animal and human studies suggested that the amygdala is involved in aggression^18–23^, the VOI was drawn on the amygdala, and a regression analysis was performed between BP_ND_ of ^11^C-cetrozole in the amygdala and aggression score. There was a positive association between aggression scores and BP_ND_ in the left amygdala in females. Earlier studies found a relationship between aggression and reactivity in the left amygdala, although one of the studies combined female and male data^21,22^. Another study reported that lower amygdala volume was correlated with more aggression in healthy females^23^. Animal studies have demonstrated that aromatase neurons in the medial amygdala regulate male aggression and maternal aggression^20^. Our results show that aromatase in the female left amygdala is associated with aggression, which indicates that estrogen synthesis in the left amygdala may induce aggression in females.

Furthermore, polymorphisms in the aromatase and estrogen receptor genes have been reported to be associated with harm-avoidance traits^24,40^. Although we did not perform genetic analyses, gene polymorphisms or DNA methylation may affect the expression of aromatase or hormone receptors. Multi-disciplinary studies that consider gene polymorphisms, epigenetic changes, and imaging are needed in the future.

Our results showed that there may be associations between aromatase in the brain and personality traits, and some of the associations may differ between sexes, while others are likely common to both. Whether sex differences exist in the brain has long been contentious and remains controversial. Classical differences in brain structure, e.g., greater corpus callosum volume in females or enlarged cortical language regions in females, have been dismissed via meta-analysis^41^. However, neurochemistry suggests tendencies for females to have higher activity in serotonergic (5-HT transporter, 5-HT_1A_ and 5-HT_2A_ receptors), dopaminergic (dopamine transporter), and GABAergic (neurotransmitter level) systems^42^, which are involved in mood, emotions, and personality traits. An animal study revealed that exogenous estrogen treatments increased the mRNA and protein levels of tryptophan hydroxylase, a rate-limiting enzyme in the production of a serotonin precursor, in the raphe nucleus of spayed rhesus monkeys^43^. Further studies are needed to clarify the differences and commonalities between sexes.

Previously the results between monoamine oxidase (MAO) level measured with PET and mood/personality disorders were reported^44,45^. Our present results also show that the aromatase level in different brain sub-regions measured with PET is associated with a variety of personality traits. The dynamics of interaction between sex hormone and monoaminergic neurotransmitter systems are partly regulated by both enzyme levels. The PET studies in combination of both enzymes may be an interesting target in the future study.

There are several limitations of the present study. The associations between TCI scores and BP_ND_ did not survive a family-wise error correction, then were analysed with a significance threshold of P < 0.001, uncorrected, k ≥ 10 voxels. As for the SON and the right amygdala of females, the associations with TCI scores were discussed even though the criteria were not fulfilled (k < 10 voxels). When we could increase the number of subjects, the results might become clearer. As regards sex hormone in plasma, we measured testosterone, free testosterone, estradiol, and progesterone in both males and females, however, free testosterone in female (N = 2), estradiol in male (N = 7) were too little to be quantified. Thus we tested the association between aromatase level and free testosterone in male or estradiol in female.

Although the subjects in this study were all healthy, there were associations between aromatase levels in the brain and personality traits, which suggests that regulation of estrogens might affect personality. Further PET studies that include patients with personality disorders using other PET tracers for estrogen and androgen receptors would help clarify the association between sex hormone systems and personality traits.

## Methods

### Subjects

Subjects were recruited by advertisements at Osaka City University and RIKEN. Twenty-four healthy adults (11 females and 13 males) participated in the present study. Participants were excluded if they had past or current serious medical illnesses and/or organic brain diseases or if they took drugs actin on the central nervous system. All females were not taking oral contraceptives and had regular menstrual cycle. Two females (35 and 45 yrs old) and 2 males (36 and 35 yrs old) received a whole-body PET scan to measure their radiation exposure, and 10 females and 11 males (average age of 34.7 ± 6.4 and 31.7 ± 8.1 yrs, mean ± SD, respectively) received a brain PET scan. Each subject completed the validated Japanese version of BAQ^25^ and TCI^27,28^ before PET scanning. All experiments were conducted in compliance with national legislation and the Code of Ethical Principles for Medical Research Involving Human Subjects of the World Medical Association (the *Declaration of Helsinki*) and registered to the UMIN Clinical Trials Registry (No. UMIN000006586). This study was approved by the Ethics Committee of the Kobe Institute of RIKEN and the Osaka City University Graduate School of Medicine. All participants provided written informed consent for participation in the study.

### Positron emission tomography

^11^C-Cetrozole was synthesized at Osaka City University Hospital according to the previously published procedure^29^. The desired compound was dissolved in a mixture of polysorbate 80 (0.1 ml), propylene glycol (0.9 ml), and saline (9 ml). The identity and concentration of ^11^C-cetrozole were assessed using high-performance liquid chromatography. The specific activity of ^11^C-cetrozole was 81.2 ± 26.0 GBq/μmol (mean ± SD) at administration. The radiochemical purity was greater than 99.5%. The dose of ^11^C-cetrozole was 4.7 ± 1.0 MBq/kg bodyweight. Subjects were positioned in the PET scanner (Biograph-16, Siemens, Knoxville, TN, USA) with their heads lightly tied with bandages to minimize movement during the scans. The left and right median cubital veins were cannulated for blood sampling and radiotracer administration, respectively. Four males received cannulation in the left radial artery instead of the median cubital vein for arterial blood sampling. Before the emission scans, CT scans were performed for head positioning and attenuation correction. At the start of the emission scan, ^11^C-cetrozole was intravenously administered for approximately 30 sec, and the catheter line was flushed with 15–20 ml saline to prevent radiotracer retention. Serial PET scanning of the brain was performed for 60 min in the 3-dimensional dynamic mode in the following frames: 6 × 10 sec, 6 × 30 sec, 11 × 60 sec, and 15 × 180 sec. Blood samples were taken 5, 10, 20, 30, 45, and 60 min after administration of ^11^C-cetrozole from the venous line and at 10, 20, 30, 40, 50, 60, 70, 80, 90, 100, 110, 120, 130, 140, 150, 160, 170, and 180 sec, and 4, 5, 10, 20, 30, 45, and 60 min after administration from the arterial line. Venous and arterial blood samples taken after 5 min and later were used for radiometabolite analyses.

### Magnetic resonance (MR) image

Fine structural whole-brain T1-weighted magnetic resonance anatomical images were acquired using the Philips Achieva 3.0 TX (Royal Philips Electronics, Eindhoven, The Netherlands) with the following parameters: Repetition time = 5.9 msec, echo time = 2.7 msec, flip angle = 12 degrees, slice gap = 0 mm, matrix size = 256, field of view = 220 mm, voxel size = 0.86 × 0.86 × 0.90 mm.

### PET image processing

Brain PET images were reconstructed by Fourier rebinning and 2-dimensional filtered backprojection without additional smoothing filters. For quantitative image analyses, PMOD software (PMOD Technologies Ltd., Zurich, Switzerland) was used. VOIs were delineated in the thalamus, amygdala, hypothalamus, and medulla, which are structures known to contain a rich supply of aromatase enzyme^5–7^, and in the cerebellar lobules on the same individual’s MR images and transferred to PET images. Decay-corrected time-activity curves were generated for each brain region, arterial blood plasma, and parent unchanged compound, as measured by thin-layer chromatography (Supplemental Fig. S1). The time-activity curves for plasma and parent unchanged fraction were fitted to a 3-exponential model and a Hill function, respectively. The data with arterial blood sampling were analysed with a Logan plot^46^, and the total distribution volume (Vt) in each brain region was calculated. The data without arterial blood sampling were analysed with a Logan reference tissue model based on average k2’^47^ using the cerebellum as a reference. Nondisplaceable binding potential (BP_ND_) and distribution volume ratio (DVR), which are linear functions of enzyme availability, were calculated (DVR = BP_ND_ + 1). Since no arterial blood sampling is preferable because of possible intense pain caused by arterial puncture, we compared the data with or without arterial blood sampling, that is, the data calculated by Logan plot or by Logan reference tissue model. Vt values of all examined brain regions were divided by the Vt value of the cerebellum and were compared with BP_ND_ values. Since the difference between normalized Vt values and BP_ND_ was 4 ± 1% (mean ± SD, N = 4), we decided to employ the Logan reference tissue model to analyse the remainder of the data. Then, BP_ND_ images were generated by model fitting. After co-registration of PET and MR images, whole-head structural images were normalized to the Montréal Neurological Institute (MNI) T1 image template, with the same parameters applied to the BP_ND_ images. BP_ND_ images were resampled to a voxel size of 2.0 × 2.0 × 2.0 mm using SPM8.

### Quantification of aromatase in the rich regions

For measurements of the aromatase level in the brain, VOIs of the amygdala, hypothalamus, thalamus, and medulla were superimposed on BP_ND_ images. Because ^11^C-cetrozole binding was heterogeneously distributed in the thalamus, the thalamus was further divided into 5 subregions, namely, anterior medial, anterior lateral, central medial, central lateral, and posterior parts, consistent with a prior study^48^. BP_ND_ values were extracted from BP_ND_ images. The difference between sexes in each VOI was analysed by a t-test. All P-values were two-tailed, and P values less than 0.05 were considered significant. These analyses were performed with the GraphPad PRISM 5.0 software package (GraphPad Software, Inc., La Jolla, CA).

### Statistical analysis of PET images

For aggression, we focused on the amygdala since earlier studies showed that this region is implicated in aggression^18–23^. A VOI of the amygdala was delineated using the WFU-Pickatlas SPM tool^49^. Using SPM8, voxel-wise analysis was performed on the amygdala of BP_ND_ images by applying the score of BAQ as a covariate. A family-wise error corrected significance threshold was set at P < 0.05 in the amygdala. The associations between scores of TCI and BP_ND_ were analysed as whole-brain analyses with a significance threshold of P < 0.001, uncorrected, k ≥ 10 voxels (=80 mm^3^). Voxel-wise analyses were performed on the BP_ND_ images using the general linear model in SPM8, with covariates of 7 traits of TCI. Although the criteria were not fulfilled (k < 10 voxels), 6 and 4 traits of TCI showed the associations in the identical coordinates (x = 8, y = 2, z = −14 and x = 24, y = −6, z = −20, respectively), thus these 2 regions were discussed separately.

## Electronic supplementary material

Supplementary Information
