# Supplementary material for: Association between aromatase in human brains and personality traits
Source: Sci Rep. 2018 Nov 15;8:16841. doi: 10.1038/s41598-018-35065-4 (PMC6237866; doi:10.1038/s41598-018-35065-4)
Supplement: Supplementary file 1 — Supplementary Information [file 41598_2018_35065_MOESM1_ESM.docx]

**Association between aromatase in human brains and personality traits.**

Kayo Takahashi^1,2^, Takamitsu Hosoya^1,3^, Kayo Onoe^1^, Tadayuki Takashima^1^, Masaaki Tanaka^2^, Akira Ishii^2^, Yasuhito Nakatomi^2,4^, Shusaku Tazawa^1^, Kazuhiro Takahashi^1^, Hisashi Doi^1^, Yasuhiro Wada^1,2^, Yasuyoshi Watanabe^1,2,*^

^1^RIKEN Center for Life Science Technologies, 6-7-3 Minatojima-minamimachi, Chuo-ku, Kobe, Hyogo 650-0047, Japan.

^2^Dept. of Physiology, Osaka City University Graduate School of Medicine, 1-4-3 Asahi-cho, Abeno-ku, Osaka 545-8585, Japan

^2^Institute of Biomaterials and Bioengineering, Tokyo Medical and Dental University, (TMDU), 2-3-10 Kanda-Surugadai, Chiyoda-ku, Tokyo 101-0062, Japan.

^4^Dept. of Metabolism, Endocrinology and Molecular Medicine, Osaka City University Graduate School of Medicine, Osaka, Japan

**Supplementary Information**

**Whole-Body Dosimetry.** Dynamic and whole-body scans were performed using an Eminence B/L PET scanner (Shimadzu, Kyoto, Japan). Five or six sequential whole-body scans with continuous bed motion were acquired. For the dosimetry assessment, volumes of interest were delineated on brain, liver, kidneys, small intestine, urinary bladder, and ovaries. PET images were converted to a standardized uptake value normalized to body weight and injected dose. Using the OLINDA/EXM software package, the absorbed dose was estimated. Effective dose was 0.00491 ± 0.00274 (mean ± SD, N = 2) and 0.00218 ± 0.00020 (N = 2) mSv/MBq in females and males, respectively.


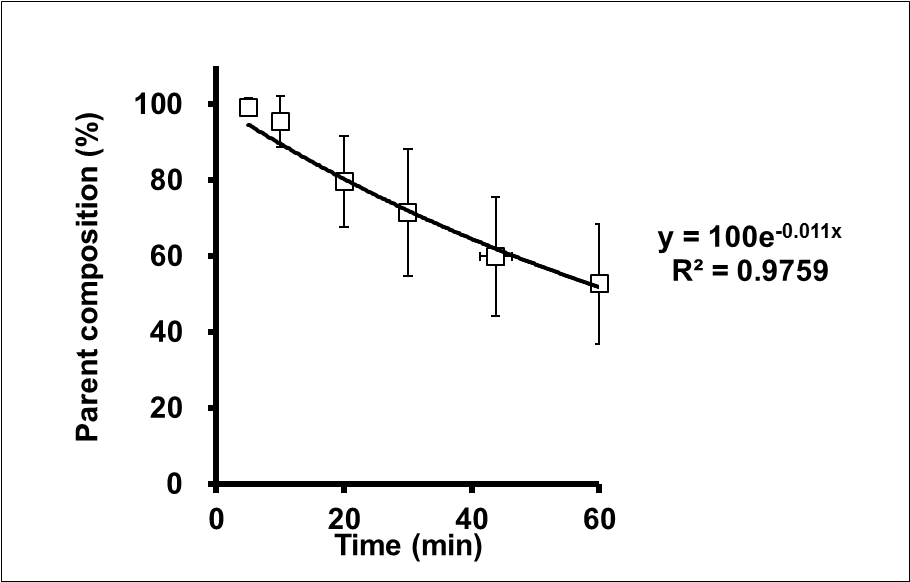


**Figure S1. Radiometabolite Analysis.** Parent composition of ^11^C-cetrozole in human plasma (mean ± SD, N = 21). Collected blood samples were deproteinated by precipitation with acetonitrile. After centrifugation, the supernatants were applied to RP-18 thin-layer chromatography (TLC) plates (Merck Biosciences, Darmstadt, Germany). Plates were developed at room temperature with acetonitrile/water/formic acid (50:50:0.75) as a mobile phase. After migration, plates were dried and exposed to BAS TR2040 imaging plates (Fuji Film, Tokyo, Japan) for 40 min. The distribution of radioactivity on the imaging plates was determined with digital PSL autoradiography using a Fuji FLA-7000 analyzer at 50-μm resolution, and the data were analyzed using the MultiGauge image analysis program (Fuji Film). The parent compound remained ca. 50% at 60 min after the administration.
